# Supplementary figures and images for: Natural variation in Arabidopsis thaliana highlights a key role of glyoxalase I;2 in detoxifying glucose-derived reactive carbonyl species
Source: BMC Plant Biol. 2025 Oct 2;25:1288. doi: 10.1186/s12870-025-07238-7 (PMC12490080; doi:10.1186/s12870-025-07238-7)

MWM

WT

K229N

F278I

K229N-F278I

(kDa)

146 -

67 -

45 -

21 -

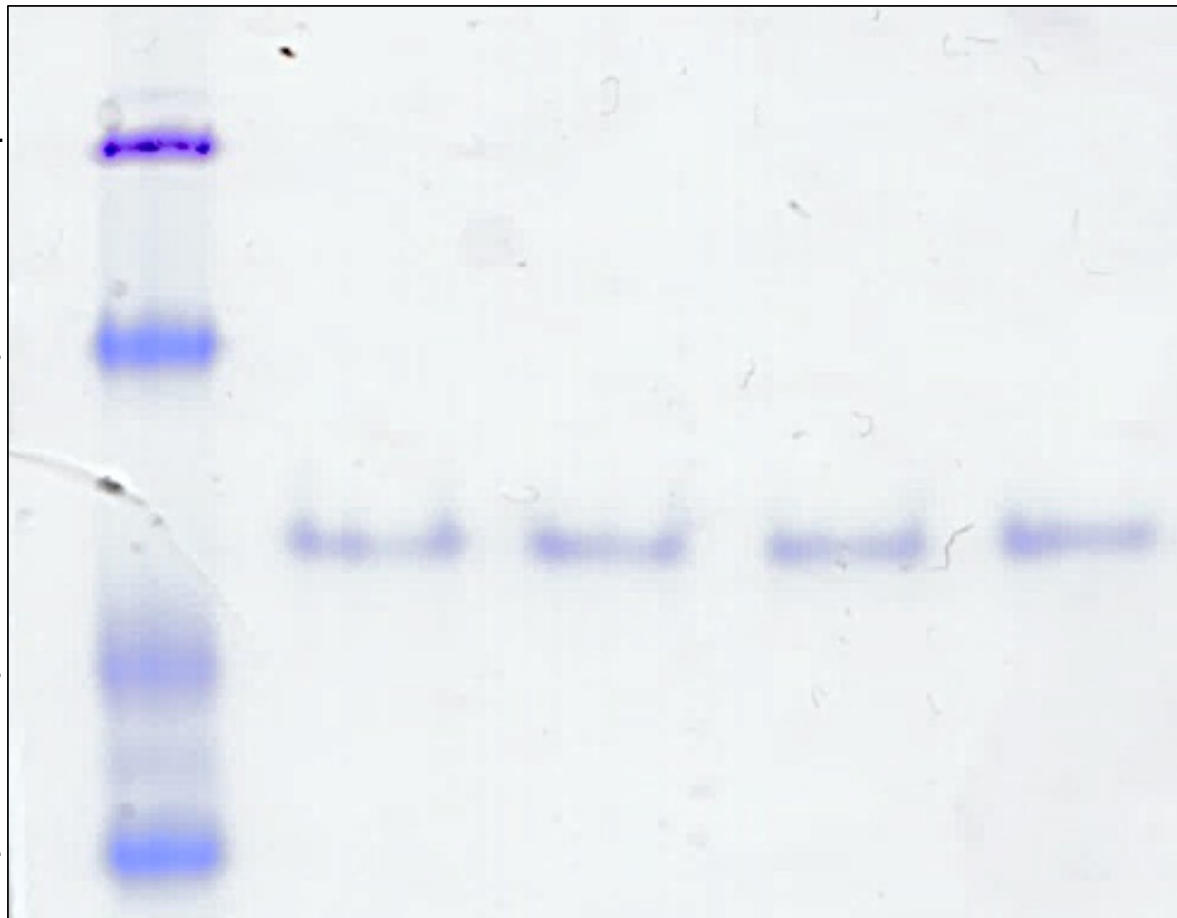

Supplement: Supplementary file 1 — Supplementary Material 1: Supplementary Figure 1. Native PAGE of GLXI;2. Two μg of recombinant GLXI;2 of Col-0 WT, and the mutants K229N, F278I, K229N-F278I were analysed in a 10% (w/v) native PAGE and visualized with Coomassie Brilliant blue. MWM: SERVA Marker Mix for Blue/Clear Native PAGE (SERVA Electrophoresis GmbH; Heidelberg, Germany) [file 12870_2025_7238_MOESM1_ESM.pdf]

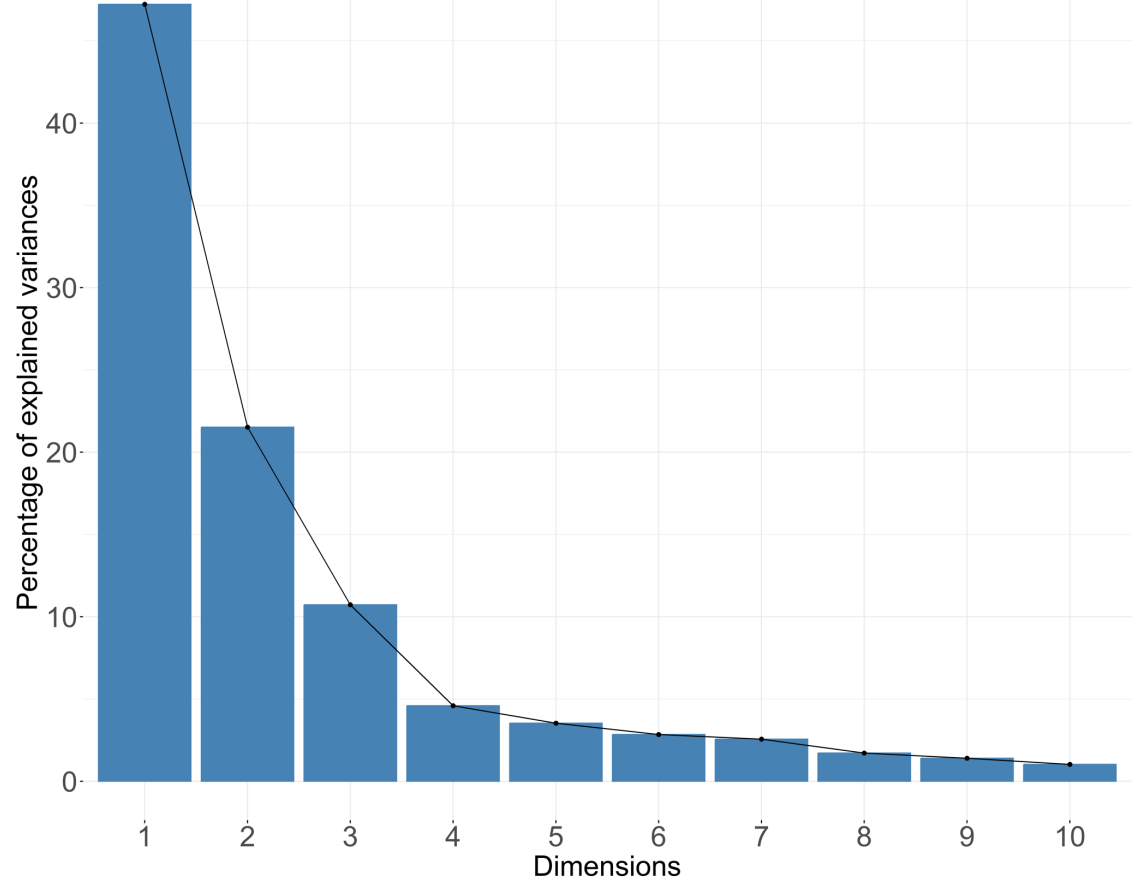

Supplement: Supplementary file 2 — Supplementary Material 2: Supplementary Figure 2. Scree plot showing the variance explained by each principal component in the PCA. The plot aids in determining the number of components to retain for subsequent analysis [file 12870_2025_7238_MOESM2_ESM.pdf]
